# Mycophenolate mofetil alters the antioxidant status in duodenum of rats: Implication for silymarin usage in mycophenolate mofetil-induced gastrointestinal disorders

**Published:** 2013

**Authors:** Sanaz Sheikhzadeh, Hassan Malekinejad, Rahim Hobbenaghi

**Affiliations:** 1 *Department of Pharmacology and Toxicology,Faculty of Veterinary Medicine, Urmia University, Urmia, Iran; *; 2 *Department of Pathology, Faculty of Veterinary Medicine, Urmia University, Urmia, Iran.*

**Keywords:** Antioxidant status, Gastrointestinal disorders, Gastro-protective effect, Mycophenolate mofetil, Silymarin

## Abstract

Mycophenolate mofetil (MMF) as an immunosuppressive agent is used to prevent graft rejection. One of the adverse effects of long time administration of MMF is the gastrointestinal disorder. This study aimed to investigate the gastroprotective effect of silymarin (SMN) on MMF-induced gastrointestinal (GI) disorders. Twenty-four adult female Wistar rats were assigned into three groups including the control and test groups. The control animals received saline (5 mL kg^-1^) and the test animals were treated with MMF (40 mg kg^-1^, orally) and saline, MMF and silymarin (SMN, 50 mg kg^-1^, orally) for 14 consecutive days, respectively. To evaluate the GI disorders due to the MMF-induced oxidative stress and subsequently the protective effect of SMN, malondialdehyde (MDA), total thiol molecules (TTM) levels and total anti-oxidant capacity (TAC) were determined. Additionally, histopathological examinations in the duodenal region of small intestine were performed. The MMF-increased level of MDA was reduced by SMN administration, while the MMF-reduced level of TTM increased significantly (*p* < 0.05) by SMN administration. Histopathological examinations showed the goblet cell reduction and congestion in the MMF-received animals; while SMN was able to improve the MMF-induced goblet cell reduction and congestion. Our data suggest that the MMF-induced GI disorders are characterized by changes in antioxidant status, which presented by the elevation of MDA level and reduction of TTM concentration. Moreover, the improved biochemical alterations and histopathologic damages by SMN indicating its gastroprotective and antioxidant effects.

## Introduction

Over the past decade, significant progress has been made in improving graft and patient survival with organ trans-plantation.^1^ Mycophenolate mofetil (MMF) is an important and commonly used drug in the maintenance of immunosuppression for recipients of all types of organ transplantation. Mycophenolate mofetil is metabolized into its active metabolite-mycophenolic acid (MPA).^2^ Subsequently MPA is extensively metabolized to phenyl mycophenolic acid glucuronide (MPAG) and MPA acyl-glucuronide (AcMPAG). The MPA and AcMPAG inhibit inosine-5' mono-phosphate dehydrogenase (IMPDH), a key enzyme to the *de novo* production of purines for T and B cells.^3^

Despite of effective immunosuppressive potency, gastro-intestinal (GI) disorder is one of the main adverse effects in patients treated by MMF.^4^ It is well known that MMF exerts toxic effects including ulcerative esophagitis, reactive gastropathy, and graft-versus-host disease (GVHD)- like features throughout the entire gastro-intestinal tract. In addition, MMF-related colitis is one of the common causes of afebrile diarrhea in transplant patients who receive MMF.^2^ It has been reported that the colonoscopic biopsy specimen frequently shows histologic features similar to those of GVHD or Crohn’s disease in MMF-treated transplant patients with persistent afebrile diarrhea.^5^^-^^7^

Previously, we showed that the MMF-induced GI disorders are mainly related to local inflammatory reactions which were highlighted with increased nitric oxide (NO) and myeloperoxidase activity in the gastro-intestinal tract.^8^ To minimize the MMF-induced side effects including GI disorders, attempts should be focused to understand the exact pathogenesis of MMF-induced damages including GI-disorders. 

Silymarin (SMN) is a polyphenolic flavanoid isolated from fruits and seeds of the milk thistle (*Silybum-marianum*).^9^ SMN is used globally as a hepatoprotective remedy in human medicine.^10^ Many gastrointestinal disorders can be treated and/or prevented by silymarin preparations.^11^ Its antioxidant, anti-inflammatory and anti-cancer effects have been also reported.^12^ In this study we aimed to investigate the antioxidant status and histopathological changes in the duodenal section of MMF-received animals. Moreover, the gastroprotective effect of SMN on MMF-altered factors was investigated. 

## Materials and Methods

**Chemicals. **Silymarin (SMN, S 0292), 5.5’-Dithiobis-2-nitrobenzoic acid (DTNB), N-(1-naphtyl) ethylendiamine. 2HCl (NED), hexadecyltrimethyl ammonium bromide, and tetramethylbenzidine N-(1-naphthyl) ethylenediamine. 2HCl were purchased from Sigma-Aldrich (Schnelldorf, Germany). Thiobarbituric acid, phosphoric acid (85%), dimethyl sulfoxide (DMSO), sodium nitrite and ethanol were purchased from Merck (Darmstadt, Germany). N-butanol was obtained from Carl Roth, GmbH Co. (Karlsruhe, Germany). Sulfanilamide was purchased from ACROS (Acros Chemical, Princeton, NJ, USA). All other chemicals were commercial products of analytical grade. Mycophenolate mofetil was provided by Hoffman La Roche (Basel, Switzerland) and was suspended in the normal saline containing 0.4% Tween 80, 0.9% benzylalcohol.^13^ SMN was dissolved primarily in small volume of ethanol (100 mg per 400 µL) and then adjusted the final volume with using saline (7.6 mL) to reach the ethanol percentage below 5% of final volume.

**Animals and Experimental Design.** Twenty four adult female Wistar rats (180-200 g) were obtained from the animal house of the Faculty of Veterinary Medicine, Urmia University, Urmia, Iran. The rats were acclimatized for one week and during adaptation and experimental periods had free access to food and water. The experimental protocols were approved by the ethical committee of Urmia University in accordance with principles of laboratory animal care (NIH publication no. 85-23, revised 1985). Animals were assigned into control and test groups (n = 8). 

Animals in the test group subdivided to following groups: A) MMF group; animals in this group received MMF (40 mg kg^-1^, orally, every day at 15:00 PM); B) SMN 50 group, animals in this group received MMF (40 mg kg^-1^, orally) and SMN (50 mg kg^-1 ^per day, orally and every day at 9:00 AM); The control group received only normal saline (0.9%, 5 mL kg^-1^) containing the same amount of the test compound solvent during the 14 days experiment period. The selected doses of MMF and SMN were based on our previous studies.^8^^,^^14^

**Tissue samples collection.** On day 15, the anesthetized rats by diethyl ether (Merck, Darmstadt, Germany), were euthanized using CO_2_ chamber and immediately the duodenal section of small intestine was dissected out and rinsed with chilled normal saline. The samples were then divided into two parts which the first part was fixed in 10% formalin in phosphate buffer saline for further pathological examinations and the second part was snap frozen in liquid nitrogen and kept at -70 ˚C till further biochemical analyses. 

**Malondialdehyde determination.** To determine the lipid peroxidation rate in the control and test groups, the MDA content of the duodenal samples was measured using the thiobarbituric acid (TBA) reaction as described previously.^15^ Briefly, 0.2-0.3 g of the samples were homogenized in ice-cooled KCl (150 mM), and then the mixture was centrifuged at 3000 *g* for 10 min; 0.5 mL of the supernatant was mixed with 3 ml phosphoric acid (1% v/v) and then after vortex mixing, 1 mL of 6.7 g L^-1^ TBA was added to the samples. The samples were heated at100 ˚C for 45 min, and then chilled in ice. After addition of 3 mL N-butanol, the samples were centrifuged at 3000 *g *for 10 min. The absorbance of supernatant was measured by Novaspec II spectrophotometer (Pharmacia Biotech, Cambridge, UK), at 532 nm and the amount calculated according to simultaneously prepared calibration curve using MDA standards. The amount of MDA was expressed as nmol per mg of protein. The protein content of the samples was assessed based on Lowery *et al*. method.^16^

**Measurement of total thiol molecules (TTM). **Total sulfhydryl level in the duodenal section was measured as described previously.^17^ Briefly, 0.3-0.4 g of the duodenal samples was homogenized in ice-cold KCL (150 mM), and the mixture was centrifuged at 3000 *g* for 10 min. 0.6 mL Tris-EDTA buffer (Tris base 0.25 M, ethylene diamine tetra acetic acid 20 mM, pH 8.2) was added to 0.2 mL of the supernatant of the tissue homogenate, and after quick vortex mixing, 40 µL 5.5’-dithiobis-2-nitrobenzoic acid (10 mM in pure methanol) was added. The final volume of this mixture was made up to 4.0 mL by an extra addition of pure methanol. After 15 min incubation at room temperature, the samples were centrifuged at 3000 *g* for 10 min and ultimately the absorbance of the supernatant was measured at 412 nm. The TTM capacity was expressed as nmol per mg of protein in samples. The protein content of the samples was measured according to the Lowry *et al*. method.^16^

**Assessment of total antioxidant capacity (TAC).** The total antioxidant capacity in the serum of control and test groups was measured. The assessment carried out based on ferric reduction antioxidant power (FRAP) assay.^18^ Briefly, at low pH which was provided using acetate buffer (300 mM, pH 3.6), reduction of FeIII-TPTZ (2, 4, 6-tri-2-pyridyl-1,3,5-triazin, Merck, Darmstadt, Germany) complex to the ferrous form produces an intensive blue color that could be measured at 593 nm. The intensity of the complex following addition of the appropriate volume of the serum to reducible solution of Fe^III^-TPTZ is directly related to total reducing power of the electron donating antioxidant. Aqueous solution of Fe^II^ (FeSO_4_.7H_2_O) and appropriate concentration of freshly prepared ascorbic acid were used as blank and standard solutions, respectively.

**Histopathological examinations.** Tissue samples, which previously had been preserved in 10% buffered formaldehyde, were embedded in paraffin and 5-6 µm sections were made by using a rotary microtome and stained with Hematoxylin and Eosin (H & E) for examination under light microscope. To evaluate the level of damages following exposure to MMF and the protective effects of SMN, indices such as goblet cells number and congestion level were scored numerically. For each animal in the test and control groups at least three slides from the duodenum were prepared and the averages of scored marks were analyzed. To count the goblet cells 30 microscopic fields were randomly chosen in the duodenal region and at the 400× magnification the cells were counted. To evaluate the congestion, the evaluation criteria were as follows: 0 for no congestion, 1 for mild congestion, 2 for moderate congestion and 3 for severe congestion. The histopathological studies were conducted by a pathologist who was unaware of the study purposes. 

**Statistical analysis. **The mean and standard deviation of the measured parameters were calculated. The results were analyzed using Graph Pad Prism software (version 2.01, Graph Pad software Inc. San Diego, California, USA). The comparisons between groups were made by analysis of variance (ANOVA) followed by Bonferroni post-hoc test. A *p* less than 0.05 was considered statistically different.

## Results

**Fig.1:**
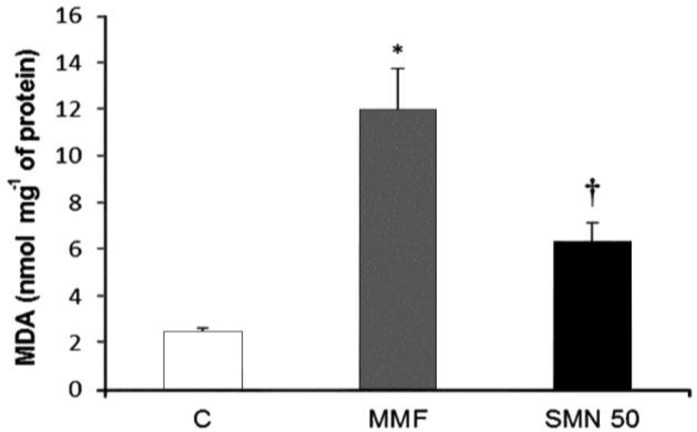
Effect of SMN on MMF-increased MDA level in duodenal region; MMF increased the level of MDA in duodenal region and SMN reduced the MMF-elevated MDA content. Data are given as mean ± SD (n = 8). C = Control; MMF = Mycophenolate mofetil received animals; SMN 50 = Animals which received both MMF and SMN.

**SMN reduced the MMF-elevated MDA content** *. *The MDA level of duodenal region following 14 days MMF administration was measured. The animals that received MMF showed a substantial increase of MDA content compared to the control group. The MDA level in the animals that received SMN and MMF, decreased significantly (*p* < 0.05) in comparison to the animals which received only MMF (Fig. 1).

* indicates significant difference (*p* < 0.05) between MMF-received group and controls, and † represents significant differences between MMF-received group and SMN-treated group.

**Fig. 2:**
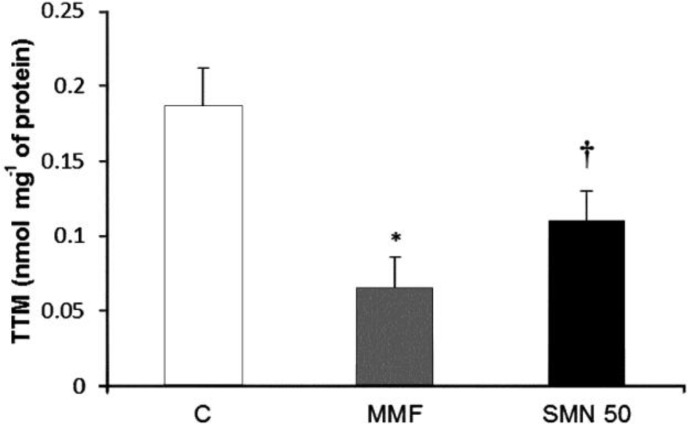
Effect of SMN on MMF-induced TTM content in the duodenal region; MMF decreased the level of TTM in duodenal region and SMN reduced the TTM depletion. Data are given as mean ± SD (n = 8). C = Control; MMF = Mycophenolate mofetil received animals; SMN 50 = Animals which received both MMF and SMN.

**Silymarin protected from the MMF-induced TTM depletion** *. *To analyze the sulfhydryl group concentration following 14 day MMF administration, the level of total thiol molecules (TTM) in the duodenal region was determined. Results showed that the TTM concentration was significantly (*p* < 0.05) reduced in the MMF-received animals, while SMN could protect from thiol molecules depletion (Fig. 2).

**Fig. 3:**
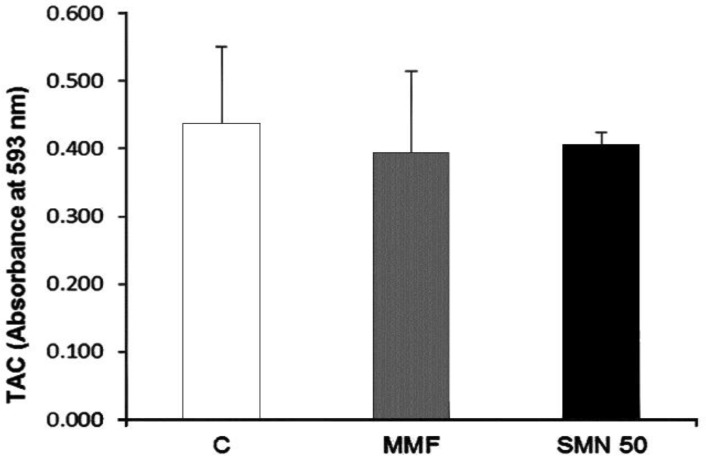
Effect of SMN and MMF on TAC level; No significant changes were observed in TAC level (*p* > 0.05). Data are given as mean ± SD (n = 8). C = Control; MMF = Mycophenolate mofetil received animals; SMN 50 = Animals which received both MMF and SMN.

**Effect of SMN and MMF on TAC level.** The total anti-oxidant capacity of animals after 14 days was determined in all groups. The results indicate that neither the MMF administration nor the SMN treatment could significantly (*p *< 0.05) change the total antioxidant power (Fig. 3).

* indicates a significant difference between MMF-received group and control group, and † represents significant differences between MMF-treated group and SMN-treated group.

**Fig. 4:**
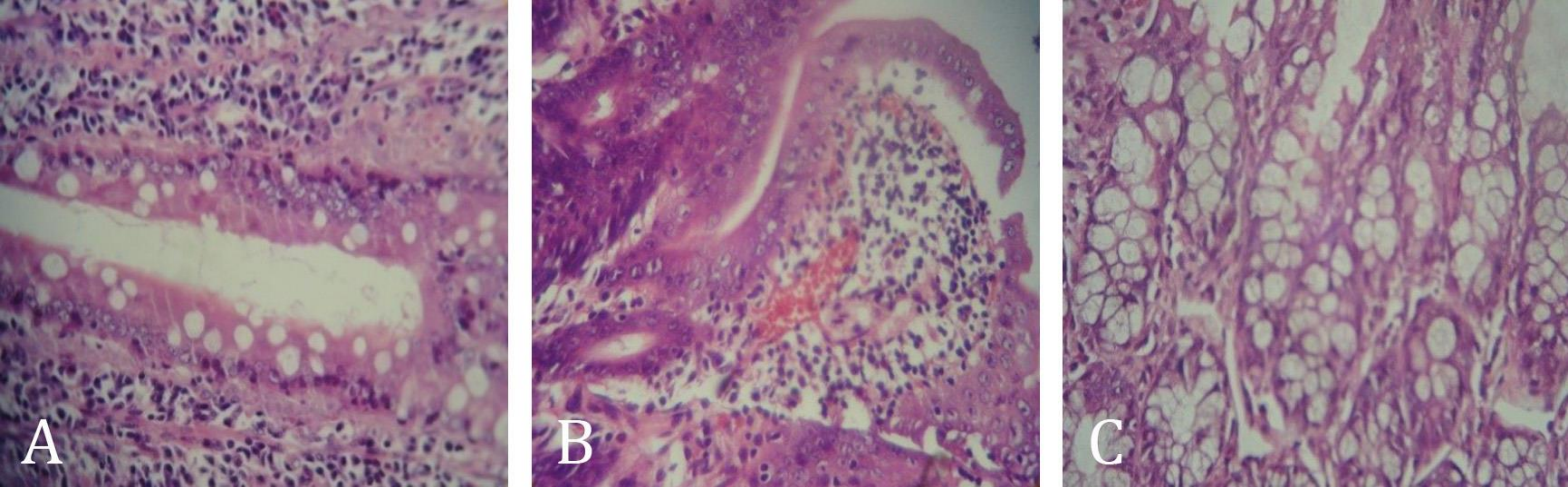
Photomicrograph of the rat’s duodenal region; (A) the control group, (B) the MMF-received group (40 mg kg^-1^), and (C) The SMN 50 group, which concurrently received SMN at 50 mg kg^-1^ dose level with MMF. The congestion and goblet cell reduction in the MMF-received group and attenuation of both pathological characters in SMN-treated group are observed (H & E, 400×).

**SMN prevented from the MMF-induced goblet cell reduction and attenuated the congestion rate in the duodenal region. **The histopathological examination of the duodenal region in the control group showed no remarkable pathologic changes (Fig. 4A). Severe congestion in villus apical section, villus atrophy and significant reduction (*p* < 0.05) in goblet cells number in the duodenal region were the pathological manifestation of MMF administration (Fig. 4B). Silymarin protected from the MMF-induced villus atrophy and goblet cell reduction. In addition, animals that concurrently received SMN with MMF, showed minor changes such as slight congestion in villus apical section (Fig. 4C). The severity of histopathological lesions as scored numerically was depicted in Table 1.

**Table 1:** Effect of SMN on the MMF-induced histopathological changes in the duodenal region (mean ± SD).

**Groups**	**No.**	**Congestion**	**No. of Goblet cells **
**Control**	8	-	20.70 ± 3.30
**MMF**	8	3.00 ± 1.30 *	2.00 ± 0.85 *
**SMN ** **50**	8	1.00 ± 0.34 †	26.00 ± 6.30 †

* indicates a significant (*p* < 0.05) difference between the control and MMF-received animals, and

† represent significant differences between the MMF-treated group and SMN-treated group.

## Discussion

The present study showed that the MMF-induced gastrointestinal disorders were reflected with clinical manifestations such as anorexia and body weight loss. Moreover, biochemical changes including MDA and TTM levels alternations and histopathological findings such as goblet cell reduction and congestion rate in lamina propria of duodenal region confirmed the MMF-induced gastro-intestinal injuries. Additionally, the current study also showed the protective effect of SMN as a natural antioxidant and anti-inflammatory compound on the MMF-induced GI disorders.

The purine synthesis in lymphocytes relies exclusively on the *de novo* pathway, which is inhibited by MMF. The anti-proliferative effect of MMF is also effective on cells other than lymphocytes, e.g. the rapidly dividing cells in the gastrointestinal tract. The epithelial cells such as enterocytes and goblet cells production depend approximately 50% on IMPDH.^19^ The direct effect of MPA on GI may attribute to its anti-proliferative effects on GI epithelial cells.

Following epithelial cells disruption pathological signs such as villous atrophy, goblet cell reduction and clinical symptoms like diarrhea are observed.^20^ In addition; previous studies confirmed the MMF exerted biphasic effects on the proliferation of human conjunctiva goblet cells (CGCs). Mycophenolate mofetil within the range of 0.25-1.00 ng mL^-1^, significantly increased the CGC proliferation, However, at higher concentrations (25-50 and 100 ng mL^-1^), decreased CGCs proliferation.^21^ Goblet cells secrete protective mucins and trefoil proteins that are required for the movement and effective expulsion of GUT contents, and provide protection against chemical damage.^22^ Therefore, the reduction of goblet cells as a consequence of MMF-administration could potentiate MMF-induced GI disorders.

The MMF-reduced goblet cells number was improved by the SMN administration, indicating protective effect of SMN on epithelial cells. The cytoprotective effect of SMN on epithelial cells against the arsenic-induced apoptosis has already been demonstrated.^23^

Congestion was another histopathological finding that were studied and we found that the MMF-received animals showed a severe congestion of duodenal region compared to the control animals likely associate to the active metabolite of MPA. Intestinal perforations, gastrointestinal bleeding, gastric and duodenal ulcers have been reported in some transplant patients on MMF therapy.^24^^,^^25^ In our study those animals which received SMN with MMF, showed slight congestion of duodenal region. It should be noted that the activated white blood cells produce free radicals in the intestinal mucosa that in turn increase the permeability of blood vessels and expand the inflammation. Release of inflammatory mediators and enzymes result in intestinal damage, bleeding and diarrhea.^26^ Anti-oxidants can prevent release of free radicals and tissue damage in the body.^27^ The results of current study indicate that SMN was effective in the elimination of intestinal inflammation and tissue damage. Silymarin with stabilizing cell membrane and enhancing the cellular glutathione concentration could possibly improve the MMF-induced congestion.^11^^,^^28^ Glutathione is responsible for detoxification of the free radicals in the body.^29^^,^^30^ Reportedly, SMN has been used to reduce the liver and kidney congestion.^31^^-^^33^

Further to the histopathological examinations, biochemical analyses, such as MDA and TTM levels in the duodenal region and TAC in the serum were determined. In the inflammatory condition, infiltration of immune cells to the site of inflammation, leads to the production of a large amount of harmful components including the reactive oxygen species (ROS) which contribute to oxidative damages in various cellular components resulting in cell death.^34^ On the other hand, lipid peroxidation is a reaction which leads to the production of free radicals such as peroxy radicals in cells and tissues. In fact, lipid peroxidation can potentiate the injuries that initiated by free radicals.^35^ The mechanism responsible for MMF-related GI disorders has not been fully elucidated yet. There are reports indicating that GI disorders may be related to mycophenolic acid (MPA) and/or its metabolite (AcMPAG).^35^ Several possible mechanisms by which AcMPAG induces gastrointestinal toxicity have been suggested in experimental studies: (1) formation of adducts with proteins or small molecules such as glutathione may contribute to drug toxicity either through direct or indirect (glutathione depletion, which was presented in this study with TTM depletion) disruption of the intestinal epithelial cell function;^37^ (2) the adducts serve as antigens with subsequent hypersensitivity that triggers immune reactions; (3) AcMPAG causes the release of inflammatory cytokines.^38^^,^^39^ The elevated level of MDA confirmed the lipid peroxidation as an inflammatory biomarker and simultaneously reduced level of TTM (as an important source of antioxidant capacity) in the duodenal region of the MMF-received animals also indicated that oxidative stress is involved in MMF-induced GI disorders. Despite a significant depletion of TTM in the duodenal section, the total antioxidant capacity in these animals remained unchanged. This may be explained by the fact that after chronic exposure to excessive amounts of oxygen radicals, the body's total thiol groups decrease, but in compensation, other types of antioxidants of the body are stimulated and thus total antioxidant capacity of the body remains normal.^40^ In addition, *de novo* is the predominant pathway for synthesis of thiol-containing compounds such as glutathione and with inhibition the *de novo* pathway by MMF administration, total thiol molecules reduced but total antioxidant capacity which is including thiol and other compounds does not significantly reduce, as reported in this study.^41^

The protective effect of SMN on MMF-induced oxidative damages was also demonstrated in this study. The antioxidant and free radical scavenging potency of silybin (as an active component of SMN) against chemical-induced lipid peroxidation have been reported.^42^ The intestinal anti-inflammatory activity of SMN in the attenuation of macroscopic colonic damages also have been previously demonstrated.^43^ Silymarin not only has free radical scavenging abilities, but also enhances the activity of anti-oxidant enzymes.^35^

In conclusion, we showed that in the MMF-induced GI-disorders, in addition of histopathological damages which characterized by goblet cell reduction and congestion, the biochemical alterations including a significant elevation of MDA content along with a remarkable reduction of TTM level were involved. This study showed that MMF-related GI disorders may attribute to its oxidative stress reactions, which may lead us to minimize these injuries with using SMN as a gastro-protective antioxidant.
